# 

**Published:** 1918-06-15

**Authors:** 


Third Annual General Meeting.

**Matrons' and Sisters' Appointments** ... .. 236

**The Higher Education of Nurses** ... ... 237

Its Relation to Applicants for Training.

**Some Annual Meetings** ... ... ... ... 239

**The Editor's Letter-Box** ... ... ... ... 240

Nervous and Mental Disorders.

**Parliament and the Profession** ... ... ... iv

**Hospital and Institutional Results** ... ... iv

**The Institutional Worker** ... ... (Suppl.) 1

A Register of Nurses' Ward Work: Answer to April Question.

RATES OF SUBSCRIPTION: Twelve months, 6/6; Six months, 4/-; three months, 2/-, post free to any address in the United Kingdom. Abroad, Twelve months, 8/8; Six months, 5/-; Three months, 2/6, post free. **THE HOSPITAL "Index" to Volume LXIII October 1917 to March 1918, is now ready, price 3½d. per copy, post free.** Address The Manager, THE HOSPITAL, "The Hospital" Building, 28/29 Southampton Street, Strand, London, W.C. 2.

**URGENT NOTICE.**

To ensure a copy of THE HOSPITAL regularly, readers must place a definite order with their Newsagent TO-DAY. After the middle of June, in consequence of the new Paper Restrictions Order, it will not be possible for Newsagents to stock copies for chance customers.

DO NOT DELAY. ORDER

**"THE HOSPITAL" TO-DAY**

and obtain your copy regularly EVERY THURSDAY.

### THE COLLEGE OF NURSING

**(Limited by Guarantee).**

### THIRD ANNUAL GENERAL MEETING.

THE third annual general meeting of the College of Nursing was held on Thursday, June 6, at the Royal Society of Medicine, the Hon. Sir Arthur Stanley, G.B.E., C.B., M.P., in the chair. Upwards of 200 nurses were present.

After congratulating Miss Dorothy Foster, R.R.C., a member of the College, on the award of the Military Medal for her splendid example of calmness and composure in supervising the transfer of the patients in one of the hospitals recently bombed by the Germans, the Chairman referred to the somewhat confident strain in which the Report was couched, which, he hoped, the members would agree was justified.

### THE THREEFOLD AIM.

**Sir Arthur Stanley**, dealing with the Report from the business side of the College, said :

There are three things that we have to aim at. The first thing is that we want to establish our Register in such a way that it will be practically a matter of course for every probationer, when she finishes her term of training and becomes a trained nurse, to enter her name upon the Register of the College. That is our first object. The second object is to raise a fund, the interest upon which will be sufficient to pay for the ordinary business running and maintenance of the College. And the third and last object—but this is one that must be postponed until after the war—is the erection of an actual building, or the purchase of a building, here in London, to serve as the headquarters of the great College of Nursing. Unless, of course, we can find some kind friend who will give us a suitable building, say such as this, that is a matter which must necessarily, as I say, be postponed until after the war. Those are the three objects that we have set out to attain, and those objects, or, rather, the manner in which we attain those objects, in London will, I hope, serve as a model to the centres which are springing up throughout the country, and about which I shall have something to say later on.

### FROM 2,553 TO 8,922.

Now, as regards the first one of those objects, the Register. I said, I think, in my remarks last year that the whole power, the whole strength of the College, its power for good and its power to influence not only the nursing profession, but to influence legislation regarding nurses, must depend entirely upon the manner in which the nurses came forward to put their names upon the Register of the College. Unless we have a large number of names upon the Register we cannot claim to speak in the name of nurses; and unless we speak really in the name of the nurses, then it is not much good having a College at all. Well, now, one of the most cheering things in the Report this year is the paragraph which relates to the very extraordinary increase in the number of nurses who have registered with the College during the last twelve months. Last year when we met, and when we had before us the Report for the year which ended March 31, 1917, it was announced in that Report that the number of nurses who had actually paid their fee—and we have learned wisdom in these matters, and do not count any name as being upon the Register until we have received her guinea—(laughter)—the number of nurses who had registered and paid their fee was 2,553, but this year, on March 31, the end of our financial year, that number had gone up to 7,962. (Applause.) Well, that is only on March 31. We got out our Report as soon as we could afterwards, a month or so afterwards, but the figure by that time had grown from 7,962 to 8,263; and even in the short time that has elapsed since we got out our Report that figure has again grown to 8,922. In other words, we have got within an appreciable distance of 9,000 members on the Register who have actually paid and have become members of the College. It will interest you probably to know that the number of applications we have had for admission to the Register of the College amounts to 11,741. Of those, of course, some have not been eligible, and some have not taken up their election for various reasons. The best reason as regards a fair number of them is because they have got married in between the time when they asked to be registered and the time when they were registered. And I am sorry to say that, of course, there have been a few vacancies caused by death. But I think when you realise that we have had getting on for 12,000 applications, and that we are now within measurable distance of having 10,000 nurses actually enrolled, you will agree that our progress in a couple of years has not been bad. (Applause.)

### SPADE-WORK BY MEMBERS.

Before I leave that subject, may I ask you to remember of what vital importance it is that all trained nurses who are eligible for the College should become members of the College? We shall, I hope, before long be presenting our Registration Bill to Parliament. It will matter, for example, when the Bill comes before Parliament, whether we have behind it a body of 20,000 nurses, 30,000, or 40,000. The greater the number is the more certain the Bill is to go through. Therefore it is not enough just to join, and to pay your guinea, and then to come up here to the meetings, or to read the accounts of it. We really do want you to undertake with us the spade-work without which we cannot possibly get the successful harvest that I am perfectly certain we are going to get.

### £8,692 ACCUMULATED.

Now, as to the second object, the endowment fund. We have made a slight alteration in our method of showing our finances. It was done at the suggestion of the accountants, in order to show an absolutely clear and simple statement of the financial position of the College. We show all our available funds, and we put them into one fund called the accumulated fund. I cannot compare it very well with last year because we showed it rather differently; we only showed the accumulated fund for the year, and that was over £800. This year we show a sum of £8,692, and that is the total accumulated funds of the College. Now, that figure is satisfactory for this reason. When we fixed the fee at a guinea our hope was that we should be able to put into an endowment fund £1 of the guinea, using the other shilling paid by the nurse for our expenses of registration, postage, etc. Now we have got at this moment 7,962 nurses, or had at the end of the financial year. But instead of only putting on one side, or in the accumulated fund, £7,962, we have been able to include even the shillings that we expected to spend on expenses. Now those 7,962 nurses

**COLLEGE OF NURSING: Meeting—*(continued).***

paying one guinea each comes to £8,360, whereas the actual money that we have got in our accumulated fund is £8,692, or £300 or more over what the nurses themselves have actually paid. The auditor, Mr. Mayhew, who has given an immense amount of trouble to the work of the College, for a much less fee certainly than would have been charged if he had been dealing with a purely business concern, wrote to me yesterday to say how very sorry he was that he could not attend this meeting, and he ends by saying this: "I should have liked to congratulate the members on the steady development of the College and its very satisfactory financial position." Now that comes from a very much higher authority than myself, and I am very glad to be able to read it to you.

THE BRITISH WOMEN'S HOSPITAL COMMITTEE.

Now it is, of course, within the knowledge of all of you that a fund has been started during this past year called the Nation's Fund for Nurses. This has been started by a splendid Committee, the Committee of the British Women's Hospital, the members of which are connected largely with the theatrical profession. These ladies thought that the best way in which they could help during the war would be by helping to collect money for such objects as they thought worthy of encouragement. And one of the first objects for which they have collected large sums of money was the Scottish Women's Hospitals, one of which hospitals, as you know, was under Dr. Elsie Inglis, who died for her country as truly as any soldier fighting at the Front. Those hospitals have done glorious work out in the Balkans and elsewhere. These ladies also, when they heard that the Red Cross were going to start a Home at the Star and Garter, Richmond, for paralysed and disabled sailors and soldiers, came forward voluntarily with an offer to find the money necessary for the erection of a building on that site. Well, I was one of the Committee who had to deal with them, and I venture to say that no Committee of men has ever been dealt with more gently, or more patiently, than we were by those ladies, because as we found that they collected money for us, so we advanced the money that we wanted from £55,000 to over £100,000. Every advance they met quite cheerfully, and invariably produced the money within about a fortnight. And, not content with that, they have also given us a large sum—over £40,000—towards the endowment of the Star and Garter, and £10,000 more for a Compassionate Fund. Well, now, when a Committee like that, of ladies like those, expressed their willingness to come forward and help this movement, which, after all, is essentially a woman's movement, we felt we had nothing to do but to accept their offer with the utmost gratitude. They have, as you know, started the Nation's Fund for Nurses. The objects of that fund are twofold—first, to provide an endowment fund for the College of Nursing; and, secondly, to provide a fund which they have called the Tribute Fund, out of which assistance can be given to any nurses who are unable by reason of sickness, of ill-health, of old age, or other cause, to continue the exercise of their profession. One of the things which has struck me very much, coming new into all this question, as I did, at the beginning of the war, has been how extraordinarily little provision has been made by the nursing profession, or by anyone else, for the relief of those members of the nursing profession, of whom, as in every other profession, there must be many, who for one reason or another fall upon evil days. There are one or two small funds, but the whole united amount of these funds comes to comparatively little—not nearly enough to deal with a subject so important as this is now, and which is certain to become so much more important after the strain of these last three and a half years. Well, these ladies of the British Women's Hospital set out to help us to collect a fund out of which assistance can be given to those nurses who need it and who deserve it. A very influential Committee has already been formed to deal with the Tribute Fund. It is called a Tribute Fund because they do not wish to feel, and they do not wish the nurses to feel, that there is anything whatever in it that can be called of a charitable nature. They want the nurses to feel, as they do themselves, that it is only a just tribute from the women of England to those nurses who have done so much for the country, who have thought so much for others that they have left no time to think for themselves.

THE PAYMENT OF A JUST DEBT.

I should like to say, and I am very sorry to have to say it, that one of the only unpleasant things that we have had to deal with at all, or that we have encountered since the College of Nursing first came into existence, was the extraordinary abuse and invective that has been showered upon these ladies from one particular quarter which happens to be opposed to the College of Nursing. One would have thought that there was, after all, nothing but good in these ladies coming forward to help some of their less fortunate sisters; and it does seem incredible to me that ladies engaged in work of that kind should have been assailed, as these have been, with the most violent invective. It is all very well to say that the nurses do not want charity. I do not think they do. But this is not charity; this is only a just debt being paid to them. It is all very well to say that when we have got State Registration, when the nurses' pay and conditions of life are up to what they ought to be, then the nurses will be able to make provision for themselves. That is all very pretty, but that is not going to help the nurse who is in need at this moment, and is in need of assistance. I feel it very strongly, and I resent it on behalf of the College, and I believe all of you will resent it—that this abuse should have been showered upon these women, who after all have only come forward to help us.

WISDOM OF THE DOUBTERS.

When I had the pleasure of speaking to you last year I told you that negotiations were in progress with the Royal British Nurses' Association, and that I had every reason to hope that these would end in amalgamation. At that time the prospect was very hopeful, but I am sorry to say that other counsels prevailed with the Royal British Nurses' Association, and that our negotiations came to nothing. I will not go into all the causes. I think that the reason for breaking off the negotiations can really best be described by a word which we have only got to know during the last few years, and that is the word "camouflage." But it does not matter for what reason they were broken off—they are at an end. I think I ought to own here that there were a good many members of the College who, when we first entered into negotiations with the R.B.N.A., were very doubtful as to the wisdom of that course. Personally I was very keen about the amalgamation, because I wanted to do everything that could be done to secure unity in the nursing profession. But I am bound to say that our subsequent experience has shown that those who were averse to the negotiations knew the subject better than I did. At all events, all negotiations are now at an end,

**COLLEGE OF NURSING: Meeting—(*continued*).**

and the fact that the College has not suffered from this is shown by the really astounding figures which I have given you of the increase of members of the College during the past three or four months since all negotiations with the R.B.N.A. have been at an end.

An Elected Council in Three Years.

There is another good thing, I think—although, as I say, I still regret that the amalgamation did not come off—there is another good thing which comes from the breaking off of negotiations, and that is this. Under the agreement with the R.B.N.A. as it stood, the Council of the united bodies that was then brought into existence was obliged to remain in office for two years. Now that we are no longer tied to the R.B.N.A. we can revert to our earlier and, I think, rather better intention, which was to make the Council of the College of Nursing elective as soon as possible. It is for that reason that you were called upon within these last few weeks to vote for members to fill the places of the first members of the Council of the College to retire. As you know, one-third retire this year. You will hear to-day the announcement of the names of those who have been elected by the nurses themselves to fill the places of those who have so retired. Each succeeding year another one-third will retire. In other words, two years hence the whole of the Council of the College will have been elected by the nurse-members of the College itself. I believe that in after years, when we look back upon the history of the College, we shall feel that this day is a red-letter day in its annals. It is not only that; it is also a landmark in the road of the nursing profession. We have tried from the beginning to fashion the College so that the nurses could make their voice heard, so that when there was any legislation going forward which affected nurses there should be a body to speak in the name of the nurses and to make itself heard. That would have been quite useless if the Council had remained a nominated Council or a co-opted Council. The only way in which the nurses' voice can really be heard is through a Council elected by the nurses themselves. I do not know exactly how other nursing associations in former days have chosen their committees. One or two that I know have chosen them in rather a haphazard way. This is the first occasion, I believe, on which the nurses have definitely chosen their own representatives, and that is why I say that when we look back upon it in after years I think it will be a red-letter day, not only in the history of the College, but also in the history of the nursing profession itself.

Still Hope of an Agreed Registration Bill.

I will conclude with just one word upon the subject of the Registration Bill. As you know, we attempted to come to an agreement with the Central Committee for State Registration, and we got so close to an agreement that I am justified in saying that there was no fundamental difference of principle or policy between us. But upon one particular point, the question of the nomination of representatives on to the Provisional Council, we did not see eye to eye, and negotiations were broken off. Since then we have gone carefully into the matter. We have felt that the question at stake was not one of principle, but really only of expediency, and therefore we have waived our objection, and we have adopted the system advocated by the Central Committee for State Registration. I believe that we have really removed the only obstacle to an agreed Bill. I have been in communication with Major Chapple, M.P., who was in charge of the Central Committee's Bill; he and I, with the help of others, have gone fully into the matter, and I hope that we shall be able—in fact, the Bill is now in print—I hope we shall be able to circulate to you before long a draft of the Bill which we shall bring before the House of Commons. Major Chapple is to approach the Central Committee for State Registration, and if they are in accord with our views, then at last we shall be able to go to Parliament with an agreed Bill. Well, I may be sanguine—I may be too optimistic in looking forward to that happy consummation; but at all events, even in these days of rationing, there is no charge made for hope. (Laughter.)

The Value of Registration.

I am afraid that some nurses at all events have been taught to look far too much to State Registration as a panacea for all their ills. State Registration, as you know, is a thing to which we attach the greatest possible importance. In fact, it is really the first plank in the College programme. But State Registration of itself can do very little unless there is behind it a strong body such as the College, which is ready to take the Act when it is passed and to use to the best advantage of the nurses the benefits that that Act confers. That is why I wish to impress upon you that, necessary as the College is now, before the Act is passed, it will be even more necessary when that Act is in existence. That is why I ask each one of you in your own way, and in your own homes, to do all you can to advance the College of Nursing in all its branches.

Local Centres Throughout the Country.

There is one subject which I have not touched upon, but which I can deal with in two words, and that is the establishment of local Centres. As you know, we told you last year that we wished to decentralise as much as was expedient, in order that every nurse, in whatever locality she lived, might feel that she was really in touch, through the Centre near her home, with the headquarters of the College up in London. Our scheme of Centres has progressed far more quickly and far better than we even dared to hope. Taking them in alphabetical order, we have already got Centres established in Birmingham, Bristol, Leeds, Leicester, Liverpool, Manchester, Plymouth, Sheffield, and Truro. And we have in course of formation, or rather are anticipating, Centres in Derby, Hampshire, and Newcastle. The work of that side has become so important that we have asked Miss Cowlin, who was assistant secretary of the College, to devote the whole of her attention to that branch of the College's work. I am glad to say that Miss Cowlin has visited most of the Centres. They were all of them delighted to see her, and, from what I have heard, they will all of them be even more delighted to see her again.

**The New Council Members.**

After the re-election of the auditors and votes of thanks to the Council and staff, the Chairman proceeded to read the names of the new members of the Council, the list of whom was handed to him by the returning officers in a sealed envelope. The names of the newly elected members are :—

English and Welsh Section.

Comyns Berkeley, Esq., M.C., M.D., F.R.C.P.

Miss Amy Hughes, late Superintendent Queen Victoria Jubilee Nurses' Association.

Miss Cox-Davies, R.R.C., Matron, Royal Free Hospital.

**COLLEGE OF NURSING: Meeting—(*continued*).**

Major-General Sir Berkeley Moynihan, C.B., M.S., F.R.C.S., Honorary Surgeon to Leeds General Infirmary.

Miss A. McIntosh, C.B.E., R.R.C., Matron, St. Bartholomew's Hospital.

Sir James Cantlie, F.R.C.S., K.B.E.

Miss E. C. Barton, R.R.C., Matron, Chelsea Infirmary.

Miss Margaret Hogg, C.B.E., Matron, Guy's Hospital.

Scottish Section.

Miss A. W. Gill, R.R.C., Lady Superintendent, Royal Infirmary, Edinburgh.

Professor Glaister, M.D., Glasgow University.

Irish Section.

Miss A. M. Curtin, Matron, Mater Infirmorum Hospital, Belfast.

Andrew Beattie, Esq., D.L., Dublin.

**The Chairman:** Well, ladies and gentlemen, I know, I think, almost all of them, and we could not possibly have a better lot elected to help us to carry on the work of the Council.

The Returning Officers' Work.

**Miss Gill:** I have much pleasure in moving a vote of thanks to the returning officers, Miss Biggar and Miss Devitt. This is the first election which has been carried out in a postal way by the College, and it has meant an enormous amount of work for them. Over 7,000 nomination papers had to be circulated, every paper had to be checked, and it must have meant a very great deal of work for the returning officers. I think the election has been very well worked, and it has stirred up an enormous amount of interest among nurses all over the country, nurses in France and elsewhere, who have filled up nomination papers, and have all been intensely interested in this election. I am sure that it is an election which has furthered the interests of the College, and made its members realise the part they are taking in the work of the College, and their own responsibility to the College, and that altogether it has done a very great deal of good. I agree with Sir Arthur Stanley that we shall look back upon this first election hereafter as a red-letter day and a landmark in the nursing profession, and I have very much pleasure in moving a vote of thanks to the returning officers.

**Miss Vincent** formally seconded the vote of thanks, which was cordially passed.

Votes of thanks were passed to the Council of the Royal Society of Medicine for the loan of their hall, and to the Chairman, Sir Arthur Stanley, on the motion of Mr. Comyns Berkeley and Miss Chaff respectively.

The Chairman in his reply, having in mind the fact that all who register with the College and pay one guinea become life members, with the view to keep intact as an Endowment Fund all the fees received from those on the Register, threw out the suggestion of a voluntary levy of 5s. each member during the first few years to cover the current expenses of each such year (£3,000).

### Matrons' and Sisters' Appointments.

Auxiliary Military Hospital, Hampton Court, Leominster.—Miss Hope Dibban has been appointed nurse-matron. She was trained at the Nottingham General Hospital; has been night sister at Beaucroft Red Cross Hospital, Wimborne; has worked on ambulance trains and at the Royal Victoria Hospital, Netley.

Binefield Auxiliary Hospital, Oxted.—Miss Edith Mabel Porritt has been appointed night sister. She was trained at the Tynemouth Union Infirmary, and has been staff nurse at the Huddersfield War Hospital and at the Manor War Hospital, Epsom.

Clare Hall Sanatorium, South Mimms, Barnet.—Miss Ada C. Barnard has been appointed sister. She was trained at London Hospital, and has since been sister at Manchester Military Hospital, at Hull Royal Naval Hospital, and has done private nursing.

Cottage Hospital, Wincanton.—Miss Frances Miller has been appointed nurse-matron. She was trained at the West Kent General Hospital, Maidstone, where she was later holiday sister; theatre and casualty sister at Royal Albert Hospital, Devonport; theatre and children's ward sister at the Hospital St. Cross, Rugby; and theatre and military sister at West Kent Hospital.

Haslemere and District Cottage Hospital.—Miss Ethel Hargreaves has been appointed matron. She was trained at Chelsea Infirmary, and has been night superintendent, ward sister, and assistant matron at the Samaritan Free Hospital for Women.

Hospital for Women, Hull.—Miss J. V. Swallow has been appointed matron. She was trained at the Northampton General Hospital, has been matron of the Hospital, Crewkerne, and of the Coldstream Hospital, and acting matron of St. Albans General Hospital.

Isolation Hospital, Biggleswade.—Miss Mary Hannah has been appointed sister. She was trained at the Mile End Infirmary and Thornton Fever Hospital, Fifeshire; has been ward sister at the Fever Hospital, Muswell Hill, and sister and acting matron at the Red Cross Hospital, Manvers House, Bradford-on-Avon. She has also done private nursing.

Jaffray Hospital, Erdington, Birmingham.—Miss Annie E. Haslam, Q.A.I.M.N.S.R., has been appointed sister. She was trained at Bagthorpe Infirmary, Nottingham, and has been staff nurse at the No. 1 War Hospital, Reading.

Mary Hewetson Hospital, Keswick.—Miss E. Alice Makins has been appointed nurse-matron. She was trained at the Liverpool Royal Infirmary; has been sister at the Liverpool Children's Infirmary and at the Southport General Infirmary, and night superintendent and assistant matron at the Edinburgh Royal Hospital for Mental Diseases.

Royal Infirmary, Stirling.—Miss E. C. Gemmill has been appointed sister. She was trained at the Borough Hospital, Birkenhead.

Royal Victoria and West Hants Hospital, Boscombe.—Miss Ada Guthrie has been appointed theatre sister. She was trained at the Victoria Infirmary, Glasgow; has been ward sister at the Military Hospital, Edmonton, and charge sister at Queen's Park Military Hospital, Blackburn.

St. Mary Islington Infirmary, Highgate Hill.—Miss Alice Kavanagh, Q.A.I.M.N.S.R., has been appointed sister. She was trained at the above institution, later being staff nurse there, and has held posts at the Shorncliffe Military Families' Hospital, and at the County of London War Hospital, Epsom.

Sanatorium, Matlock.—Miss Amy Week has been appointed sister-housekeeper. She was trained at Leeds General Infirmary and Hull Sanatorium, and has since been senior sister and matron at Birmingham Skin Hospital, temporary matron of Birmingham Eye Hospital, of Sutton Coldfield Cottage Hospital, matron of Princess Helena College, Ealing, and has done private nursing.

The Hospital, Bridge Street, Paisley.—Miss Christmas Auld has been appointed sister. She was trained at the Shoreditch Infirmary and the Fever Hospital, Falkirk, and has been charge sister at the Shoreditch Infirmary and at the County Hospital, Motherwell.

**NOTE.—Particulars of Appointments for insertion in this column will be welcomed.**

### Some Annual Meetings.

### Belgrave Hospital for Children.

THE fifty-first annual report of the Belgrave Hospital for Children, Clapham Road, states that of the 647 patients cared for last year 146 were under one year of age and 243 were the children of sailors and soldiers; they were drawn from all parts of South London. New out-patients to the number of 8,539, whose attendances totalled 34,775, had also received attention. Expenditure had increased to £5,636—£727 more than in 1916. Whereas 84 more in-patients and 2,281 more out-patients were treated in 1914 than in 1917, the work was carried out at a cost of £1,250 less. Income last year amounted to £5,586, and, although there had again been a reduction in annual subscriptions, donations had reached their normal total.

### St. John's Hospital.

THE Earl of Chesterfield occupied the chair at an ordinary general meeting of subscribers to St. John's Hospital for Diseases of the Skin, the in-patient department of which is at Uxbridge Road, out-patients being treated in Leicester Square. It was stated that the increased cost of requirements, with no corresponding advance in income, as well as a steadily increasing overdraft at the bank, had compelled the Board to curtail the work and close the children's ward and one women's ward, thus reducing the number of beds available from forty to twenty-eight. Further, it was pointed out that such a step had been necessitated at a time when the number of beds available in London for skin diseases was totally inadequate. Expenditure had exceeded the income for 1917 by £257, and would have been considerably more had it not been for a sum of £400 received from the trustees of the "Annie Zunz" Fund. In-patients admitted last year numbered 164, and out-patients 7,384 with 36,302 attendances.

